# Genetics of Long COVID: Exploring the Molecular Drivers of Persistent Pulmonary Vascular Disease Symptoms

**DOI:** 10.3390/idr17010015

**Published:** 2025-02-13

**Authors:** Sana Ayyoub, Navneet Kaur Dhillon, Olga Tura-Ceide

**Affiliations:** 1 Department of Medical Sciences, Faculty of Medicine, University of Girona, 17004 Girona, Spain; sanayyoub@outlook.com; 2 Division of Pulmonary and Critical Care Medicine, Department of Internal Medicine, University of Kansas Medical Center, Mail Stop 3007, 3901 Rainbow Blvd, Kansas City, KS 66160, USA; ndhillon@kumc.edu; 3 Department of Cell Biology and Physiology, University of Kansas Medical Center, Kansas City, KS 66160, USA; 4 Translational Research Group on Cardiovascular Respiratory Diseases (CAREs), Girona Biomedical Research Institute (IDIBGI-CERCA), Martí i Julià, Hospital Park Building M2, 17190 Salt, Spain; 5 Biomedical Research Networking Centre on Respiratory Diseases (CIBERES), 28029 Madrid, Spain

**Keywords:** endothelial dysfunction, long COVID, genetic polymorphism, pulmonary disorder, pulmonary hypertension, SARS-CoV-2, thrombosis

## Abstract

**Background/ Objectives:** Long COVID or post-acute sequelae of SARS-CoV-2 infection (PASC) are symptoms that manifest despite passing the acute infection phase. These manifestations encompass a wide range of symptoms, the most common being fatigue, shortness of breath, and cognitive dysfunction. Genetic predisposition is clearly involved in the susceptibility of individuals to developing these persistent symptoms and the variation in the severity and forms. This review summarizes the role of genetic factors and gene polymorphisms in the development of major pulmonary vascular disorders associated with long COVID. **Methods:** A comprehensive review of current literature was conducted to examine the genetic contributions to pulmonary complications following SARS-CoV-2 infection. Studies investigating genetic polymorphisms linked to pulmonary hypertension, pulmonary thromboembolism, and pulmonary vascular endothelialitis were reviewed and summarized. **Results:** Findings show that specific genetic variants contribute to increased susceptibility to pulmonary vascular complications in long COVID patients. Variants associated with endothelial dysfunction, coagulation pathways, and inflammatory responses have been implicated in the development of pulmonary hypertension and thromboembolic events. Genetic predispositions influencing vascular integrity and immune responses appear to influence disease severity and progression. **Conclusions:** Understanding these mechanisms and genetic predispositions could pave the way for targeted therapeutic interventions to alleviate the burden on patients experiencing long COVID.

## 1. Introduction

Members of the coronavirus have been mainly linked to mild respiratory symptoms resembling the common cold, until the emergence of the severe acute respiratory syndrome (SARS) in 2002, which was associated with more severe symptoms and a mortality rate of 13% [1]. The respiratory system is known to be the primary target of coronavirus, including the severe acute respiratory syndrome coronavirus 2 (SARS-CoV-2), due to the abundance of angiotensin-converting enzyme 2 (ACE2) receptors, particularly in the upper airway, which serves as the main entry site for this virus.

Although SARS-CoV-2 is primarily a respiratory virus, it can affect multiple organs and contribute to organ damage through various mechanisms, including direct infection of the organ, promoting a hyperinflammatory state, oxidative stress, and inducing endothelial dysfunction (ED), among others [2,3,4]. The various symptoms experienced by infected patients can persist for weeks or even months after viral clearance. This condition, commonly referred to as post-acute sequelae of SARS-CoV-2 infection (PASC) or long COVID, has been associated with over 200 estimated symptoms [5], affecting 1 in 10 patients according to a study in the USA [6].

Considering that the respiratory system is the main target of SARS-CoV-2, persistent respiratory symptoms are common, with some studies reporting continuous pulmonary function impairment and lung fibrosis in severe cases [7,8]. Dyspnea, chest pain, and cough have been reported as the most prevalent respiratory symptoms in post-hospitalized patients, affecting 37%, 16%, and 14%, respectively [9]. Pulmonary vascular symptoms, including pulmonary hypertension and pulmonary thrombosis, among others, which were attributed to inflammation, oxidative stress, mitochondrial dysfunction, DNA damage, and hypoxia [10], are also common and can be life-threatening in some cases.

This review will mention some of the genetic polymorphisms associated with long COVID, focusing on common pulmonary vascular symptoms associated with COVID-19, exploring the molecular mechanisms involved in their pathogenesis, with a particular emphasis on genetic factors that may explain the variation in symptom severity and manifestation among individuals. Also, the role of *ACE2* and *TMPRSS2* in COVID-19 severity will be discussed, considering their significant role in the SARS-CoV-2 pathogenesis.

Thus, the primary objective of this review is to provide an understanding of the genetic polymorphisms associated with long COVID, which can contribute to variations in symptoms and severity among individuals, particularly focusing on their role in the development of pulmonary vascular complications. This might aid in a deeper understanding of long COVID and the role of genetics, offering insights for future research and potential therapeutic strategies.

## 2. Role of *ACE2* and *TMPRSS2* Polymorphism in COVID-19 Severity

The SARS-CoV-2 is composed of a lipid envelope surrounding a single-stranded RNA. Embedded within this lipid envelope are key structural proteins, including the envelope (E), membrane (M), nucleocapsid (N), and spike (S) protein, which is composed of two subunits (S1, S2). Within the S1 subunit is the receptor binding domain that interacts directly with the ACE2 receptors. After binding to ACE2, a host surface protease called TMPRSS2 cleaves the spike protein at the junction between the S1 and S2 subunits. This cleavage exposes fusion peptides in the S2 subunit, allowing the viral envelope to merge with the host cell membrane and creating an entry point for the viral RNA to enter the cell [11]. A detailed review of the mechanism of SARS-CoV-2 cell entry is described by Jackson. et al. 2015 with visual representations that can aid in a better understanding of the process, and readers are recommended to refer to it [12].

Multiple receptors play key roles in SARS-CoV-2 infection and entry into cells, but ACE2 and TMPRSS2 are the most critical, facilitating viral attachment and subsequent membrane fusion. In a study analyzing approximately 81,000 genomes, researchers identified unique genetic variations in these two genes across different populations, where an *ACE2* polymorphism (p.Arg514Gly) in African/African-American populations was linked to cardiovascular and pulmonary conditions, and a prevalent polymorphism in *TMPRSS2* (p.Val160Met) was associated with an increased risk of severe COVID-19, especially in male patients and those with cancer. The study suggests that these genetic differences may help explain variations in COVID-19 severity and could inform personalized treatments [13].

A study performed on 300 patients in Pakistan reported the association of *ACE2* rs2285666 SNP with increased susceptibility to the virus, particularly in older males, while no significant association was found for *TMPRSS2* SNPs in the cohort [13]. In another study conducted on 317 patients in Egypt, no significant link was found with the *ACE2* rs2285666 polymorphism, while the *TMPRSS2* rs12329760 polymorphism, specifically the T allele and CT/TT genotypes, was associated with a lower risk of severe COVID-19, which suggests that *TMPRSS2* variations may play a protective role in disease severity [14].

Li et al., 2022, wrote a detailed review on the role of *ACE2* and *TMPRSS2* mutation and polymorphism in COVID-19 susceptibility and disease progression; following an analysis of 33 studies, 10 SNPs and 21 mutations in the *ACE2* gene, as well as 13 SNPs and 12 variants in the *TMPRSS2* gene, were potentially linked to COVID-19 [15].

These findings highlight the importance of understanding genetic variations in *ACE2* and *TMPRSS2*, as they may contribute to differences in COVID-19 susceptibility and severity across populations, and this can offer valuable insights for developing targeted therapies and personalized treatments.

## 3. Genetic Polymorphisms Associated with Long COVID

The complexity of the PACS of COVID-19 suggests that different mechanisms and molecular pathways play a role in the development of these symptoms. One possible explanation for the variation in the symptoms and intensity experienced by the patients is genetic variations. It is known that variation in a particular gene leads to differences in the phenotype among individuals. These polymorphisms have been investigated in long COVID and can explain in part the variation in responses to SARS-CoV-2 infection among individuals.

Although most studies have focused on *ACE2* and *TMPRSS2* polymorphism given their critical roles in viral entry and initial infection and subsequent symptoms, other research has examined polymorphisms in genes associated with thrombosis, immunity, and inflammation, which are particularly relevant to long COVID. In many cases, significant relationships have been identified between these gene polymorphisms and the severity and type of symptoms, suggesting a genetic predisposition to prolonged illness in some individuals.

One study examined the role of single nucleotide variants (SNVs) in influencing COVID-19 severity, particularly in those with pre-existing conditions, such as coronary artery disease (CAD). Using data from the CARGENCORS study, which included 818 hospitalized COVID-19 cases and 1636 non-hospitalized controls, the researchers identified 14 new SNVs linked to CAD, thrombosis, and inflammation that were significantly associated with severe COVID-19. A meta-analysis with the SCOURGE and UK Biobank cohorts confirmed five key SNVs associated with severe outcomes, suggesting that these genetic markers may increase the risk of severe COVID-19, which could guide targeted preventive measures and treatments [16].

Table 1 (below) showcases some of the studies carried out on genetic polymorphism and their association with the development of long COVID.

## 4. Pulmonary Vascular Disorders in COVID-19

Pulmonary vascular disorders refer to conditions affecting the blood vessels within the lung causing disturbance in the blood flow, of which several disorders have been associated with COVID-19. These include pulmonary thromboembolism, pulmonary hypertension, and pulmonary endothelialitis, among others.

The endothelium, which is the innermost layer of blood vessels, is composed of a single layer of endothelial cells that are currently known to play a significant role in maintaining vascular homeostasis through the release of various mediators, ensuring proper tissue functioning throughout the body [25].

SARS-CoV-2 can enter these cells primarily through ACE2 receptors, inducing ED, which is a well-known component in COVID-19, contributing significantly to the development of pulmonary vascular disorders by increasing vascular permeability, inflammation, and promoting a prothrombotic state, which can disrupt normal blood flow in the lungs [2,26]. These can contribute to conditions, like pulmonary hypertension, where elevated pressure in the pulmonary arteries results from vascular remodeling, and pulmonary thromboembolism, caused by clot formation and impaired blood circulation.

### 4.1. Genetics of Pulmonary Vascular Symptoms in COVID-19

**Pulmonary thrombosis:** One of the most critical vascular symptoms observed in COVID-19 is thrombosis, including pulmonary embolism (PE), which was estimated to affect about 15% of the patients [27]. Clots forming in the pulmonary vessels can form in situ within the lungs or travel from other areas of the body, such as the deep veins of the legs, causing life-threatening events by reducing blood flow, impairing oxygen exchange, and potentially damaging lung tissue [28].

A combination of hyperinflammation, endothelial injury, and coagulopathy drives the process by which COVID-19 induces PE. The infection triggers a “cytokine storm”, releasing pro-inflammatory cytokines, like IL-6 and TNF-α, which activate the coagulation cascade and inhibit fibrinolysis. SARS-CoV-2 can also induce endothelial dysfunction by direct infection, exposing the subendothelial tissue that promotes clotting. COVID-19 is also associated with elevated D-dimer levels and platelet activation, leading to the formation of thrombi. Hypoxia from severe COVID-19 exacerbates this process by increasing blood viscosity and upregulating prothrombotic genes. Furthermore, complement system activation amplifies endothelial damage and thrombus formation [29,30,31].

Studies have been conducted analyzing the genetic factors contributing to PE among COVID-19 patients, with one case study reporting a link between death from PE and heterozygous mutations in *MTHFR* and *SERPINE1* genes [32]. Another study conducted on 25 individuals with PE in Italy reported a significant association between *ACE* D/D polymorphism and an increased risk of PE in patients with severe COVID-19 pneumonia. The study found a higher prevalence of the D/D genotype in patients with PE (72%) compared to those without thromboembolic complications (46.5%), which suggests genetic susceptibility to thromboembolism in COVID-19, potentially linked to higher levels of ACE and angiotensin II [33].

In a cross-sectional study that included 140 patients, genetic polymorphisms were assessed in *F2* (prothrombin), *F5* (factor V Leiden), and *MTHFR* (methylenetetrahydrofolate reductase), along with clinical parameters, like D-dimer levels and thrombotic events. Results showed that severe COVID-19 cases had a significantly higher frequency of heterozygous mutations in all the studied genes, with the *FV* R506Q mutation carrying the highest risk for severe disease progression. Elevated D-dimer levels were also more common in patients with certain mutations (*FV* R506Q, *FV* R2H1299R, and *F2* G20210A), indicating an association between inherited thrombophilia and severe COVID-19 outcomes [34].

**Pulmonary hypertension:** Another common COVID-19 sequela is pulmonary hypertension (PH), where the blood pressure is elevated in the pulmonary vessels due to constriction of the small pulmonary artery leading to pulmonary arterial hypertension (PAH) or obstruction of the vessels due to clots causing chronic thromboembolic pulmonary hypertension (CTEPH). both of which can be fatal [35,36,37]. Pulmonary hypertension is estimated to occur in about 22% of COVID-19 patients [38].

Studies have reported similarities between COVID-19-induced pulmonary vasculopathy and PAH, including features, such as medial hypertrophy, smooth muscle cell proliferation, and genetic alterations influencing disease severity, which suggests that PH could be a long-term sequela of COVID-19 [39,40].

The genetic drivers of PH have been investigated, where one study investigated the molecular connections between COVID-19 and PAH by integrating protein–protein interactions and transcriptomic data from patients and identified shared molecular mechanisms and transcriptional regulators common to both diseases, including *RUNX1*, *HIF1A*, *JUN*, *STAT1*, *STAT3*, and *NFKB1* [41].

Bioinformatics analyses and machine learning techniques identified *SELE* and *CCL20* as key factors involved in immune and inflammatory responses. Specifically, they are associated with the activation of immune cells, like dendritic cells and T cells, which are crucial in both COVID-19 and PH. This connection suggests that the immune response triggered by COVID-19 could potentially worsen PH through the activity of these genes [42]. Another bioinformatics analysis of a GWAS dataset for respiratory failure in COVID-19 patients identified eight genes (*GATA4*, *ID2*, *MAFA*, *NOX4*, *PTBP1*, *SMAD3*, *TUBB1*, and *WWOX*) by mapping single nucleotide polymorphisms (SNPs) to nearby genes and performing protein–protein interaction (PPI) analysis. These genes formed the largest connected network and were further validated through a literature review, revealing their associations with pulmonary and cardiac diseases, including PH [43].

CTEPH is a significant yet uncommon complication of COVID-19, as it was estimated to occur in about 1.2% of patients in a UK national surveillance study [44]. Several studies have reported the association between different genetic polymorphisms and susceptibility to CTEPH development but without specifically relating it to post-COVID. One study reported that Factor V Leiden and prothrombin G20210A appear to contribute differently to the development CTEPH compared to PE alone and suggested that younger patients with PE who carry the Factor V Leiden mutation may be at a reduced risk of developing CTEPH [45].

A genome-wide association study (GWAS) conducted on CTEPH alone highlighted several important genetic contributions to the disease, including a significant association between CTEPH and a SNP within the *ABO* blood group gene, confirming that non-O blood types are a known risk factor for CTEPH development. Also, significant genetic variants were found in fibrinogen and coagulation factor XI, which are crucial in the coagulation cascade, as well as a significant genetic overlap between CTEPH and patients with PE or deep vein thrombosis (DVT). A novel finding from the study was the identification of a significant SNP near the *HLA-DRA* gene, which is implicated in immune response [46], though these findings were not linked with SARS-CoV-2 infection.

Another study identified ten key genes as highly relevant to CTEPH progression, including *IL-1B*, *CXCL8*, *CCL22*, *CCL5*, *CCL20*, *TNF*, *IL-12B*, *JUN*, *EP300*, and *CCL4*, which are linked to inflammatory responses, thrombus formation, and endothelial cell dysfunction. Also, genes involved in the NF-κB and AP-1 transcription factors, along with the TNF signaling pathway, were shown to play a significant role in CTEPH [46].

**Pulmonary endothelialitis:** Comorbidities, especially vascular disorders, like hypertension, diabetes, and coagulopathies, are strongly linked to the severity and mortality of COVID-19. This may be due to the virus’s impact on the vascular endothelium, leading to endothelialitis. By directly infecting endothelial cells through ACE2 receptors, the virus promotes inflammation, which can cause cell necrosis and disrupt coagulation [47,48,49]. Several studies mentioned the role of genetic polymorphisms in developing ED in COVID-19. For example, a study reported that the 4G/5G PAI-1 promoter polymorphism influences ED in COVID-19 patients. Specifically, the 4G4G genotype was associated with higher *PAI-1* levels, suppressed fibrinolysis, and increased NFκB activation, contributing to ED. In contrast, the 5G5G genotype posed a risk due to inflammation-induced overactivation of the fibrinolytic system [50].

To manage the hyper-inflammatory response observed in COVID-19, using immunomodulatory agents has been suggested. Agents, such as corticosteroids, IL-6 receptor antagonists, and JAK inhibitors, have shown promise in reducing the hyperinflammatory state associated with severe COVID-19 [51,52,53]. Additionally, targeting specific pathways, like NFκB activation, implicated in endothelial damage, could further reduce the risk of thrombotic and vascular complications [54,55].

#### Genetic Mechanisms of Polymorphisms in Pulmonary Vascular Symptoms

Gene polymorphism represents variation in the DNA sequence of a specific gene, which can lead to changes in protein structure, function, and/ or expression levels. These changes may disrupt normal cellular processes, including enzymatic activity, receptor binding, or signaling pathways. For example, polymorphisms in coagulation-related genes, like *F5* or *SERPINE1*, may enhance thrombotic activity by altering the protein’s susceptibility to regulation, contributing to a hypercoagulable state [56]. Similarly, variations in genes affecting vascular homeostasis, such as ACE, can increase angiotensin II levels, leading to vasoconstriction, inflammation, and fibrosis [57]. These downstream effects can exacerbate ED, promote vascular remodeling, and elevate the risk of developing pulmonary vascular disorders in certain conditions, like COVID-19.

In the case of COVID-19, polymorphisms in *ACE2* and *TMPRSS2* significantly influence susceptibility to SARS-CoV-2 infection and its downstream effects on pulmonary vasculature. For example, variants in *ACE2*, such as rs2285666, can increase ACE2 expression, enhancing viral entry into cells and leading to increased inflammation and an imbalance in the renin–angiotensin system, favoring elevated angiotensin II levels. The resultant vasoconstriction, oxidative stress, and fibrosis exacerbate ED and vascular remodeling, contributing to pulmonary hypertension [58,59]. Similarly, polymorphisms in *TMPRSS2*, such as rs12329760, can alter protease activity, either facilitating viral activation or modifying immune responses [60,61]. Such changes can intensify endothelial damage and amplify thrombotic risks, driving pulmonary vascular complications.

On the other hand, inflammatory gene polymorphisms, such as those in *IL6* (e.g., -174G>C) or *TNF* (-308G>A), can lead to excessive or prolonged cytokine production during COVID-19 infection [62,63]. These polymorphisms enhance the transcriptional activity of pro-inflammatory cytokines, driving a cytokine storm that increases vascular permeability and endothelial injury. The heightened inflammatory state can disrupt vascular homeostasis, contributing to the progression of pulmonary endothelialitis and chronic vascular inflammation.

Also, polymorphisms in coagulation-related genes, such as *F5* (R506Q) and *F2* (G20210A), can increase susceptibility to thromboembolic events by enhancing pro-coagulant activity or reducing anticoagulant regulation. In COVID-19, these polymorphisms synergize with the hyperinflammatory state to drive excessive clot formation in pulmonary vessels, leading to pulmonary thromboembolism [34,64].

### 4.2. Genetic Link Between Pre-Existing Respiratory Illnesses and Long COVID

It is important to note that preexisting pulmonary conditions, such as asthma and chronic obstructive pulmonary disease (COPD), which are known to be associated with increased inflammation and immune dysregulation, may amplify the effects of SARS-CoV-2. Genetic polymorphisms influencing immune and inflammatory responses have been linked to COVID-19 severity and may partly explain this exacerbation [65].

A shared genetic link between these respiratory conditions and COVID-19 severity and persistence has been widely explored, indicating a complex relationship between both. For example, polymorphisms in *IL6*, such as -174G>C, are associated with elevated pro-inflammatory cytokine levels, contributing to asthma severity, and prolonged inflammation in long COVID [66]. Similarly, variants in the *ADRB2* gene, like Gln27Glu and Arg16Gly, are linked to airway hyperresponsiveness in asthma and may exacerbate respiratory symptoms in long COVID due to altered smooth muscle function [67]. HLA alleles, such as HLA-DRB1*04, play a role in immune regulation and have been implicated in asthma and severe COVID-19 through dysregulated immune responses [68]. However, it should be noted that other contradictory results have been observed suggesting a protective effect of asthma on COVID-19 severity, as one study reported that individuals with a genetic predisposition to asthma may have a lower risk of contracting SARS-CoV-2 and experiencing severe COVID-19 outcomes, which could be due to a compensatory immune mechanism in these patients during the initial stages of COVID-19 [69].

In COPD, one study analyzed the association between the disease and COVID-19 and reported several key genes that could play a role in worsening symptoms during COVID-19, which could serve as potential targets for developing drugs to mitigate complications in COVID-19 [70]. Another study investigated the shared pathways involved in the comorbidity of COVID-19 and COPD, identifying critical genes, such as *CCL2*, *MMP9*, *IL1A*, and *HIF1A*, which play pivotal roles in immune responses, angiogenesis, and inflammation. Bioinformatics analyses revealed that cytokine-cytokine receptor interactions, chemokine activity, and IL-17 signaling pathways are central to the progression of both diseases. The study highlights dexamethasone, estradiol, progesterone, and nitric oxide as potential therapeutic agents, supported by their ability to modulate key inflammatory and angiogenic processes. This provides valuable insights into the molecular mechanisms underlying COVID-19 and COPD comorbidities and suggests promising directions for future research and therapeutic interventions [71].

Thus, considering the shared genetic alternations between certain respiratory illnesses and long COVID, we should note that identifying these polymorphisms in individuals with preexisting pulmonary illnesses could provide valuable insights into their risk for persistent symptoms and inform personalized therapeutic strategies.

## 5. Interplay with Environmental Factors and Comorbidities

It is worth noting that the manifestation and severity of pulmonary vascular disorders in COVID-19 are not solely attributed to genetic polymorphisms but are significantly affected by environmental exposures and comorbid conditions. For example, environmental factors, like air pollution, smoking, and occupational exposures, may exacerbate ED by increasing oxidative stress and systemic inflammation, thereby amplifying the effects of genetic predispositions [72,73]. Similarly, comorbidities, including obesity, diabetes, and hypertension, which are known to impair vascular function, may synergize with genetic susceptibilities to worsen the outcomes. Individuals with obesity may experience heightened ACE2 expression in adipose tissue, further facilitating viral entry, which may promote severe pulmonary vascular symptoms [74,75]. Recognizing the interplay between genetic and environmental factors can help us in better understanding the disease and its management.

## 6. Conclusions and Recommendations for Future Research

Considering that coronaviruses have recently been associated with severe diseases in humans, it is important to understand the pathology and molecular drivers behind their different symptoms, as these viruses are expected to emerge again in the future. Here, we focused on vascular pulmonary disorders, as these are one of the main targets of the virus and the severity of the symptoms linked to it. Understanding the role of genetic variation in the severity and the symptoms associated with the viral infection is crucial in paving the way to knowing the cause of the variation and developing more effective, personalized treatment that can improve patients’ outcomes.

Future research should prioritize large-scale genomic studies to identify novel genetic variants associated with pulmonary vascular disorders in COVID-19 and validate existing findings across diverse populations. Functional studies are essential to determine the mechanisms by which specific polymorphisms influence protein function and pathways. Developing genetic screening tools could identify high-risk individuals early, facilitating preventive strategies. Furthermore, integrating genomics, transcriptomics, and proteomics techniques with environmental and clinical data will be crucial for understanding the complex interplay between genetic and non-genetic factors. This integrated framework will enhance our ability to predict outcomes and inform the development of novel therapeutic targets and public health interventions.

By highlighting these interactions and implications, the scientific community can pave the way for more comprehensive approaches to understanding and managing pulmonary vascular disorders, ultimately improving patient outcomes in COVID-19 and beyond.

## Figures and Tables

**Table 1:** Genetic polymorphisms associated with long COVID.

Gene Polymorphism	Participants	Key Finding(s)	Reference
*ACE2* rs2285666 *ACE2* rs2074192 *TMPRSS2* rs12329760 *TMPRSS2* rs2070788	293 individuals previously hospitalized with COVID-19, of which 257 had long COVID, from 3 hospitals in Madrid, Spain.	These polymorphisms have been previously associated with COVID-19 severity, but do not seem to contribute to the development of long COVID among hospitalized patients. Only the G allele of *TMPRSS2* rs2070788 was linked to an increased risk of developing post-COVID dyspnea and gastrointestinal complications.	[17]
*ACE2* rs2106806 *ACE2* rs6629110	29 participants, of which 16 had long COVID in Madrid, Spain.	The A allele in the rs2106806 and the T allele in the rs6629110 variant were associated with increased long COVID-19 susceptibility.	[18]
*LZTFL1* rs10490770 *LZTFL1* rs11385942 *LZTFL1* rs17713054 *NADSYN1* rs12785878 *PLXNA4* rs1424597 *IL10* rs1800896 *ACE2* rs2285666 *PEDS1* rs6020298 *IL10RB* rs8178562	260 patients, of which 49 had long COVID from a hospital in Bangkok, Thailand.	All these polymorphisms were significantly associated with the development of long COVID, although the *IL10RB* rs8178562 GG genotype was associated significantly with a reduced long COVID risk.	[19]
*IL6* rs1800796 *IL10* rs1800896 *TNF* rs1800629 *IFITM3* rs12252	293 individuals, previously hospitalized with COVID-19, of which 117 had post-COVID pain, from 3 hospitals in Madrid, Spain.	Polymorphisms in these genes were associated with inflammatory response and were not associated with post-COVID-19 pain.	[20]
*ApoE* rs429358 *ApoE* rs7412	287 individuals, previously hospitalized COVID-19 patients with at least 1 post-COVID symptom, from 3 hospitals in Madrid, Spain.	*ApoE* ε4 was associated with higher SARS-CoV-2 infection risk and disease severity, but no significant association was observed between long COVID and the *ApoE* genotype (*ApoE* ε2, *ApoE* ε3, *ApoE* ε4).	[21]
*MBL2* rs1800450 *MBL2* rs1800451 *MBL2* rs5030737	387 individuals, who had COVID-19, of which 177 had long COVID, from Brazil.	No significant association was observed between long COVID and *MBL2* polymorphism, though the homozygous genotype (OO) was more prevalent in patients with severe COVID-19.	[22]
*F5* rs6025 *F2* rs1799963 *MTHFR* rs1801133 *IL6* rs1800795 *TNF* rs1800629 *MTHFR* rs1801133	226 patients, of which 101 had severe symptoms, recruited from hospitals in Brazil.	Only *MTHFR* polymorphism was associated with disease severity, which was linked to persistent symptoms.	[23]
*ACE1* rs1799752	288 previously hospitalized COVID-19 patients with at least 1 post-COVID symptom, from 3 hospitals in Madrid, Spain.	Polymorphism does not contribute to the development of long COVID.	[24]

## Data Availability

No new data were created or analyzed in this study. Data sharing is not applicable to this article.
